# PELVIC INJURY IN CHILDHOOD: WHAT IS ITS CURRENT IMPORTANCE?

**DOI:** 10.1590/1413-785220162403157540

**Published:** 2016

**Authors:** MARÍA ROXANA VIAMONT GUERRA, SUSANA REIS BRAGA, MIGUEL AKKARI, CLAUDIO SANTILI

**Affiliations:** 1. Irmandade da Santa Casa de Misericórdia de São Paulo, Department of Orthopedics and Traumatology, São Paulo, SP, Brazil.; 2. Faculdade de Ciências Médicas da Santa Casa de São Paulo, São Paulo, SP, Brazil.

**Keywords:** Fractures, bone, Pelvic Bones, Accident prevention, Child

## Abstract

**Objective:**

The purpose of this study was to assess the importance of pelvic fractures in childhood by analyzing epidemiological characteristics and associated injuries.

**Methods:**

This is a retrospective study performed between 2002 and 2012 at two trauma referral centers in São Paulo. We identified 25 patients aged 16 years old or younger with pelvic fracture.

**Results:**

The main mechanism of trauma was traffic accident (80%), followed by fall from height (16%). At hospital admission, 92% had traumatic brain injury and 40% had hemodynamic instability. Besides pelvic fractures, 56% of the children had other associated injuries (genitourinary, abdominal, vascular, chest and neurological), and 79% of them required operative treatment. According to the Torode and Zieg classification, the majority of cases were types III and IV. Seventy-two percent of all pelvic fractures were treated by surgery; 52% involved external fixation and 20% involved open reduction and internal fixation.

**Conclusions:**

The pelvic fractures in childhood can be considered a marker for injury severity, because the associated injuries usually are severe, needing operative treatment and leading to a high mortality rate (12%)*. Level of Evidence IV, Case Series.*

## INTRODUCTION

Pelvic fractures in children are rare, accounting for 2.4-7.5% of all cases of reported trauma.^1^ ^-^ ^4^ Perhaps due to its rarity, there are still no specific classification protocols and medical conduct for its aproach.^5^

Children's hip has some peculiarities as compared to the adult's.^2^ ^,^ ^5^ ^,^ ^6^ Elasticity due to the cartilaginous structures of the pelvis requires a higher energy trauma to cause a fracture. Therefore, before the occurrence of the fracture itself, other systems of the body are injured.^2^ ^,^ ^3^ ^,^ ^5^ Furthermore, the bone remodeling capacity is higher than in the adult, but the development of progressive deformities is higher in children, when the growth plate is involved in the injury.^5^ ^,^ ^6^ Unlike adult's, children's pelvic fractures do not occur with significant bleeding, probably due to increased vasoreactivity and greater adherence of the periosteum in children, which would tampon local bleeding.^1^ ^,^ ^2^ ^,^ ^4^

Due to the high energy involved in the trauma, the resulting pelvic fractures serve as potential indicators of associated lesions, as well as the severity of the trauma. The main injuries found are head trauma, chest and abdominal injuries.^1^ ^,^ ^2^ ^,^ ^5^ ^-^ ^7^ There is a strong association between childhood trauma and pelvic fracture by traffic accidents, especially trampling.^1^ ^,^ ^6^ ^,^ ^8^

The most commonly used classification of pediatric pelvic fracture is that by Torode and Zieg,^9^ which has been used since 1985. Four types are described: type I, fractures of bony prominences in avulsion; type II, iliac crest fractures; type III, simple fractures of the pelvic ring without instability and type IV, complex fractures of the pelvic ring with instability.^10^ This classification has a good correlation with trauma energy, associated injuries, type of treatment and outcome. Due to the stability and absence of associated injuries, type I fractures can be treated as outpatients. Type II and III fractures require hospitalization, usually for treatment of associated injuries. The condition of patients with type IV fractures is usually severe, involving multiple organ injury and life-threatening systems.^2^ ^,^ ^3^ ^,^ ^8^ ^-^ ^10^

It is controversial in the literature which would be the best treatment for pelvic fractures in childhood. Due to the high elasticity of a child's pelvis and its remodeling capacity, many authors suggest conservative management with traction and immobilization. In stable fractures this approach have good results with good consolidation.^3^ ^,^ ^11^ However, regarding non-operative treatment of unstable fractures there is shortage of long-term information, especially regarding deformities.^12^

The current trend is fixating fractures surgically in order to avoid complications, such as chronic pain and deformities.^5^ ^,^ ^11^

Mortality, in general, is lower in children than in adults with pelvic fracture, death resulting from associated injuries.^2^ ^,^ ^4^ ^,^ ^5^ Whatever the type of fracture, about 52% of patients require assistance to move and in their daily activities after hospital discharge.^5^ Complications, such as difference in length between limbs, functional deficit, deviations in the lumbar spine and consequent chronic low back pain are avoided by maintaining the symmetry of the pelvic ring during follow up.^12^

The objective of this study is to evaluate the importance of pelvic fractures in childhood from the analysis of epidemiological characteristics and associated injuries in two major referral trauma care centers in São Paul, SP, Brazil.

## MATERIALS AND METHODS

This is a retrospective study, held between 2002 and 2012 in two major referral centers in trauma care in the metropolitan area of ​​São Paulo *(Emergency Service at Hospital Central da Santa Casa de Misericórdia de São Paulo and Hospital Geral de Guarulhos).* We Identified 25 patients with pelvic fracture aged 3-16 years (mean = 11.04 years), 60% male and 40% female. The study was approved by the Ethics Committee under protocol CAAE 37869314.0.0000.5479.

Through analysis of medical records the following data were collected: gender; age at the accident; trauma mechanism; transportation method from the accident site to the emergency service and the elapsed time; patient assessment at admission for hemodynamic stability, traumatic brain injury (using the Glasgow Coma Scale - GCS), type of pelvic fracture and its treatment, associated injuries (thoracic, abdominal, neurological, genitourinary and orthopedic) and their treatment; need for blood transfusions; duration of hospital stay; admission at the intensive care unit (ICU); and death.

The data was summarized in a Microsoft Office Excel chart and analyzed using the statistical software EpiInfo.

## RESULTS

**Figure 1:**
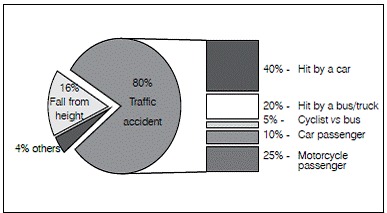
Trauma mechanism of pediatric pelvic fractures.

The main mechanism of trauma observed was traffic accidents (80% of cases), followed by falls from height (16%) and injury caused by heavy object falling into the lower limbs (4%). Among traffic accidents, the most common type was running over by car (40%), followed by accidents involving motorcycle passenger (25%), running over by a bus or truck (20%), accidents involving car occupants (10%) and cyclists hit by bus (5%). (Figure 1)

Patients were brought to the emergency service through a rescue service in 22 cases (88%). The average time between the accident site and the hospital was 36 min. At admission, 10 cases (40%) had hemodynamic instability. It was observed that 92% of patients had traumatic brain injury (TBI), 28% moderate or severe, according to the Glasgow Coma Scale (GCS <13).

**Figure 2:**
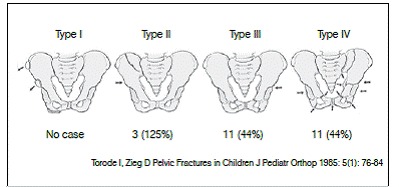
Classification according to Torode and Zieg. Figure also shows respective amount and percentage of cases found for each type of fracture.

Pelvic fractures were grouped according to the classification proposed by Torode and Zieg. Three cases (12%) were type II, 11 cases (44%) type III, and 11 cases (44%) type IV. (Figure 2) Of all pelvic fractures, 72% were submitted to surgical treatment, 52% were treated with external fixation and 20% with open reduction and internal fixation with plate and screws.

**Figura 3:**
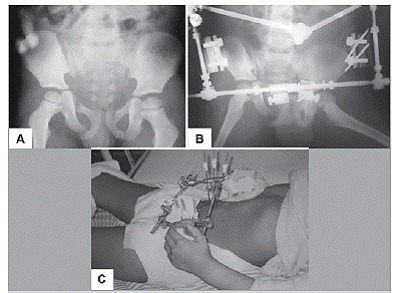
(A) Patient admitted with pelvic ring fracture (Torode and Zieg Type IV). (B) Patient was submitted to stabilization and external fixation of the hip, besides exploratory laparotomy and colostomy, x-rays and clinical image (C).

Besides the pelvic fracture, 56% of children had other associated lesions. (Table 1) Among these lesions, 11 (79%) required at least one surgical treatment (1 craniectomy, 1 trepanation, 1 vaginal tear suture, 10 exploratory laparotomies, and in some cases nephrectomy, diaphragmatic injury suture, mesosigmoid suture, hollow viscera suture, cystostomy, segment colectomy, and colostomy).

**Table 1:** Injuries associated to pelvic fracture.

Injuries	Nº of cases	% of total
Genitourinary	8	32%
Orthopedic (other)	8	32%
Abdominal	7	28%
Neurological	7	28%
Thoracic	4	16%
Vascular	2	8%

Among the genitourinary lesions we observed: urethra and bladder injury, bladder hematoma, hematuria, extraperitoneal bladder injury, ureter partial section, retroperitoneal hematoma in zone II, vulvar hematoma and genital laceration.

Among abdominal injuries, we found: liver laceration, traumatic acute pancreatitis, perisplenic hematoma, acute peritonitis, rectal injury, sigmoid injury, retroperitoneal hematoma in zones I, II and III and mesosigmoid rupture.

As neurological injuries we observed: injury of the parietal lobe, diffuse cerebral edema, subdural hematoma and sciatic nerve injury. Among chest injuries we observed: pulmonary contusion, hemothorax, hemopneumothorax and diaphragmatic giant hernia, and as vascular lesions, complete rupture of renal vessels, and partial rupture of inferior gluteal vessels.

Regarding other orthopedic injuries concomitant to pelvic injuries, we found eight cases (32%) as follows: fracture of the jaw, skull base, cervical vertebra (C3), scapula, elbow, distal third of the radius, wrist, femur, tibia, calcaneus and hip dislocation. Of these eight cases, three (37.5%) required surgical treatment.

The average hospital stay was 15.8 days (1-81 days), and nine patients (36%) required intensive support, staying on average 9.5 days (2-22 days) in the ICU. Twelve cases (48%) required blood transfusions.

The mortality rate was 12% (three children). It was found out that these children were brought to the emergency service within 30 minutes and that at admission they had hemodynamic instability, unstable pelvic fractures (Torode and Zieg type IV) and severe associated injuries (all three patients with vascular injuries, one with neurological injury and one with genitourinary injury).

## DISCUSSION

Pelvic fractures in childhood are rare occurrences in pediatric trauma.^1^ ^-^ ^4^ ^,^ ^12^ Our study identified only 25 cases treated at two large referral centers in trauma care in the metropolitan area of ​​São Paulo, SP, Brazil, in a 10 year period.

Among the traffic accidents, the most common type was running over by a car (40%), consistent with data from literature.^2^ ^,^ ^4^ ^,^ ^7^ ^,^ ^8^ ^,^ ^13^ ^,^ ^14^ Furthermore, it was found that accidents involving motorcycle passengers were the second most common mechanism (25%), despite the Brazilian legislation that determines the minimum age for a motorcycle passenger is seven years old. Surprisingly, it is worth reporting here that in half of the cases it was in fact a teenager who drove the motorcycle, even though the law prohibits driving under 18 years old.^15^ ^,^ ^16^

The average time between the trauma and the patient's admission to a hospital was 36 minutes, but three patients (12%) came by their own means, not by a rescue service. Two of three patients arrived at the hospital 48 hours after trauma due to persistent pain and both had a low-energy trauma, causing stable pelvic fractures.

All cases with hemodynamic instability on admission were involved in high-energy trauma. The high-energy trauma mechanisms were more likely associated with blood transfusion, ICU admission, presence of more severe pelvic fractures (Torode and Zieg types III and IV), presence of related injuries, and higher mortality rate as compared to low energy trauma. All these associations are consistent with the literature, however none was statistically significant.^1^ ^,^ ^3^ ^,^ ^4^ ^,^ ^8^

It has also been shown by other authors that there is an association between pelvic fracture and the presence of severe associated injuries (as hemothorax, pneumothorax, intracranial bleeding, bowel injury), which can both cause early death as lead to late sequelae if not treated promptly. This association was present in this study, but was not statistically significant.^1^ ^,^ ^3^ ^,^ ^8^ ^,^ ^12^ ^,^ ^13^

The distribution of the types of fractures according to Torode and Zieg ^10^ differs from that found in the literature. Most of the articles describe injury type I (bone avulsion) as very rare, type II and III as the most common injuries, and type IV with a lower frequency than types II and III.^3^ ^,^ ^4^ ^,^ ^10^ ^,^ ^14^ In contrast, Niedzielki et al.^17^ observed in their study that avulsion fractures were the most frequent and occurred mostly during sports training. In our study, where cases involving running over and motorcycle passenger accidents were frequent, we found that most cases were type III (44%) and type IV (44%), therefore, 88% of children had severe pelvic fractures.

In addition to this classic classification by Torode and Zieg,^9^ which is based solely on radiographs, Shore et al.^18^ proposed a change in classification, subdividing type III into two groups: type III-A including simple and stable fracture of the anterior pelvic ring, while type III-B includes stable anterior and posterior pelvic ring fractures. However, this subdivision was not considered in this study because all cases involving fracture of the anterior and posterior pelvic ring presented with instability, therefore, we had no cases that would qualify as type III-B.

Regarding treatment, most orthopedic surgeons advocate surgical treatment of pelvic fractures in childhood, especially unstable fractures, since children may have unfavorable outcomes (functional pain, pelvic asymmetry, and discrepancy of the lower limbs) with conservative treatment.^6^ ^,^ ^10^ ^,^ ^19^ ^,^ ^20^ However, other studies showed that nearly all cases were not treated surgically, only unstable fractures were operated.^13^ ^,^ ^14^ In this study, the surgical treatment prevailed (72%), including both external fixation (52%) and open reduction and internal fixation with plates and screws (20%). Most children who underwent external fixation had unstable fractures. Patients treated with open reduction and internal fixation were hemodynamically stable at admission and had GCS level 15.

Among the cases studied, 56% had associated injuries, mostly genitourinary, abdominal and neurological injuries, whereas other studies report as main injuries traumatic brain injury (neurological), chest and abdominal injuries.^1^ ^,^ ^2^ ^,^ ^6^ ^,^ ^7^ Most injuries from this study (79%) underwent surgical treatment, especially laparotomy. Every associated injury, except for one (vulvar hematoma associated with pubic fracture) occurred in high-energy trauma.

The literature mentions the need for pediatric ICU to, on average, 50% of patients, and generally because of associated injuries.^1^ ^,^ ^3^ ^,^ ^14^ This study, despite showing less need for pediatric ICUs (36%) - similar to studies by Chia et al.^8^ (33%) and Banerjee et al.^13^ (28%) - reported higher hospitalization periods - on average 9.5 days *versus* 5.5 days^8^ and 6 days. ^13^ The causes of ICU admission were also due to the concomitant presence of injuries, especially abdominal, neurological and chest injuries.

Results obtained in this study are in accordance with most other studies. Specifically, pelvic fracture, *per se*, is not a main cause of death, but associated lesions can lead to death.^1^ ^-^ ^3^ ^,^ ^7^ The mortality rate was somewhat higher when compared to other series. However, three deaths occurred in patients with serious injuries associated with hemodynamic instability, with unstable pelvic fractures. All these patients underwent laparotomy, which revealed other injury sites with abundant blood loss.

Therefore, it is important upon diagnosing a pelvic fracture in children or adolescents with immature skeleton, a strict and active evaluation of other organ systems is made, since these injuries may be responsible for severe condition, hemodynamic instability and even death.

One final point to be discussed would be the imprudence and neglect of children and adolescents. Since pelvic fractures in childhood are predominantly caused by traffic accidents, most traumas could be avoided if basic preventive measures were taken, such as keeping children away from the streets and avenues; constant supervision when not at home; using appropriate measures to transport children in vehicles such as seat belts, traveling in the rear car seat, and not transport them as motorcycle passengers. We should create awareness among parents on the importance of respecting the age limits set by law, preventing teenagers to drive motorcycles with children as passengers. It is suggested that measures to improve road safety through the implementation of activities, national legislation and educational campaigns are necessary.

## CONCLUSION

Pelvic fractures in childhood tend to be a marker of severity, since the associated injuries are usually severe, mostly requiring surgical treatment and contributing to a high mortality rate (12%). For them to occur, a high-energy trauma is necessary, mainly caused by traffic accidents (80%).
